# Positive attitudes, positive outcomes: The relationship between farmer attitudes, management behaviour and sheep welfare

**DOI:** 10.1371/journal.pone.0220455

**Published:** 2019-07-31

**Authors:** Carolina A. Munoz, Grahame J. Coleman, Paul H. Hemsworth, Angus J. D. Campbell, Rebecca E. Doyle

**Affiliations:** 1 Animal Welfare Science Centre, The University of Melbourne, Melbourne, Victoria, Australia; 2 Faculty of Veterinary and Agricultural Science, The University of Melbourne, Melbourne, Victoria, Australia; Humboldt-Universitat zu Berlin, GERMANY

## Abstract

This study examined the relationships between the attitudes and the management behaviour of the farmer and the on-farm welfare of their ewes. To our knowledge, this is the first study investigating these relationships in extensive sheep farming systems. Thirty-two sheep farmers and 6200 ewes were sampled across Victoria, Australia. Questionnaire interviews and on-farm animal welfare assessments were conducted. The ewes were assessed at two-time points, mid-pregnancy and weaning. To examine relationships between farmer and ewe variables, categorical principal component analyses, correlations and logistic regressions were used. The main findings of this study indicate relationships between farmer attitudes and management behaviour, consistent with findings from other more intensive livestock industries. Farmers were more likely to check the body condition of their ewes (Odds ratio = 2.37, *P* = 0.03), perform ultrasound pregnancy diagnosis (Odds ratio = 1.16, *P* = 0.02) and test for egg count before deworming sheep (Odds ratio = 2.88, *P* = 0.01) if they perceived these activities were important/valuable. In addition, farmers that performed these activities had a more active management style, and ewes in better welfare: fewer lame ewes at mid-pregnancy (r = -0.38 *P* = 0.04), and fewer ewes in need of further care at mid-pregnancy and weaning respectively (r = -0.47, *P* = 0.01; r = -0.50, *P* = 0.01). When combining the qualitative and quantitative analyses, behavioural attitudes (attitudes towards specific management behaviours) and perceived behavioural control (perceived barriers to performing the behaviour) emerged as the two main drivers underpinning farmer management behaviour. The results of this study indicate that the way farmers manage their ewes influences welfare outcomes, and management decisions are influenced by attitudes towards management practices. These findings demonstrate the opportunity to create change in farmer management behaviour and improve sheep welfare via targeted education programs.

## Introduction

Extensive sheep production systems allow sheep to live in a more natural environment and to perform an extensive amount of natural behaviours. Under these systems, and when resources are available, sheep have more choice in, and control over their day to day activities, such as grazing, ruminating and socially interacting with conspecifics [1,2], all of which can be indicative of good sheep welfare [3]. Although behaviour is not restricted, extensive farm systems create other environmental risks to welfare such as the possibility of predation, variable quantity and quality of feed and water and climatic extremes [2]. While welfare issues in extensive systems can be exacerbated by the environment, management practices play a significant role in mitigating these risks [4].

Improved management practices to increase sheep welfare and productivity are widely available for farmers [5–8]. However, farmer adoption of best practice is limited and warrants further investigation. For example, survey data suggest that despite training only 32% of farmers either measure the body condition of their ewes or would be willing to do so, and only 47% of farmers did or would be willing to allocate pregnant ewes to different groups according to nutritional needs [9]. More recently, a broad industry survey on 600 sheep farmers identified that only half of producers use ultrasound pregnancy diagnosis regularly, with only 31% of them separating ewes into different mobs according to nutritional needs [10]. Farmers are the key players in improving sheep welfare and farm productivity because they provide the actual care of animals and make the management decisions on their farm. Thus, a better understanding of farmer attitudes towards sheep and sheep management and barriers to best practice is imperative.

Training and education programs for farmers usually focused on knowledge transfer (e.g. nutrition management, pasture management, etc.), however, it has been demonstrated that programs should also focus on generating change in the beliefs and attitudes that underpin behaviours to create a long-term change [11]. This present study examined how farmer attitudes influence their management behaviour, and how management influence welfare outcomes for the animal. In order to do this, we used the Human-Animal Relationship Model (HAR) by Hemsworth and Coleman [12]. It was hypothesised that farmer attitudes will relate to management behaviour and management behaviour will influence the on-farm welfare of extensively managed ewes.

**Fig 1:**
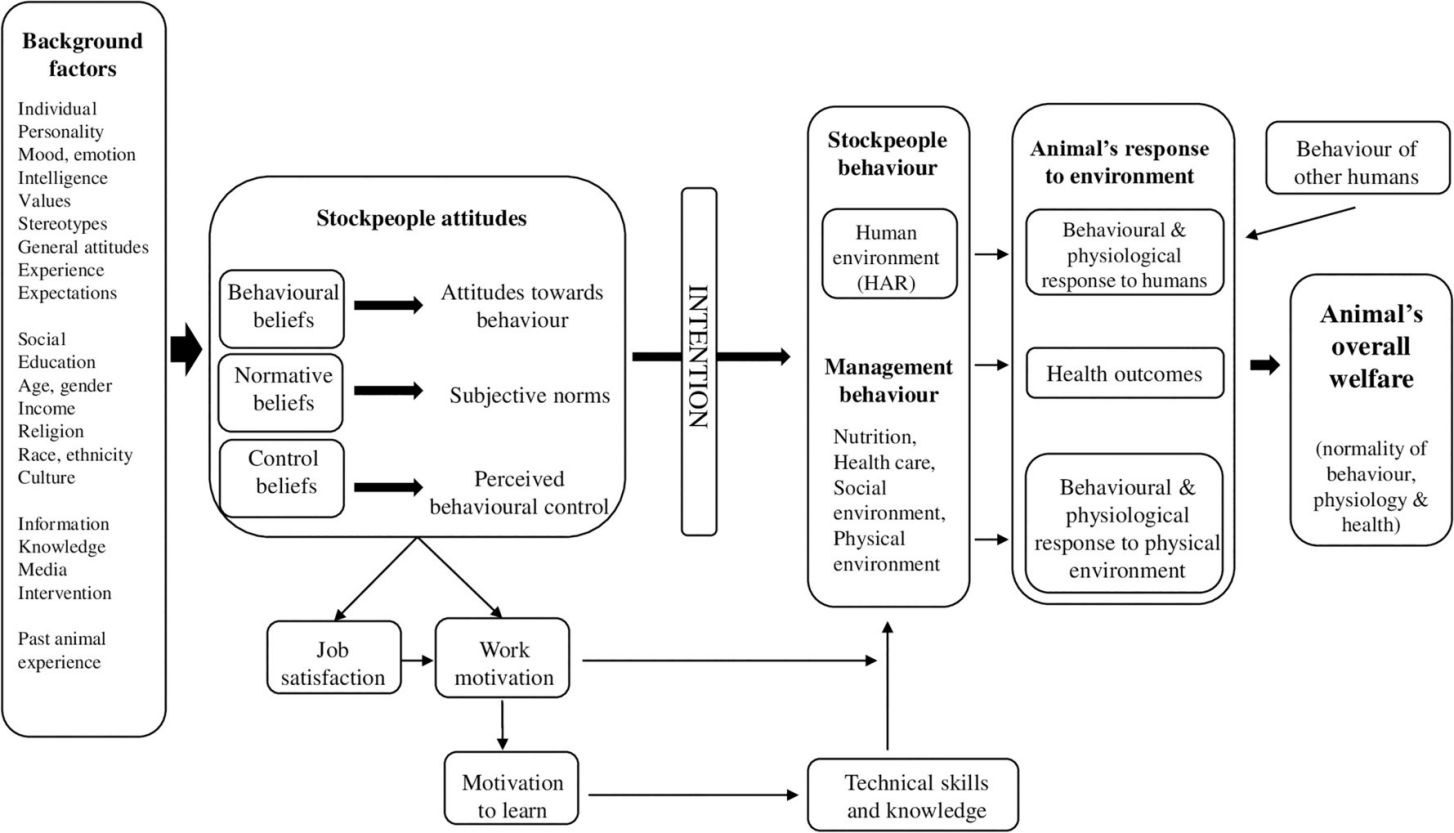
The Human-Animal Relationship Model based on the Theory of Planned Behaviour adapted from [14].

The HAR Model (Fig 1), is based on the Theory of Planned Behaviour [13], and establishes that stockperson behaviour is likely to be determined by three ‘key drivers’ (1) attitudes towards the behaviour, which refers to a person’s beliefs of the outcome (favourable or unfavourable) of a specific behaviour, (2) subjective norms, which refers to a person’s beliefs about other people expectations (e.g., parents, spouse, friends) or social pressures to perform a specific behaviour and (3) perceived behavioural control, which refers to a person’s beliefs about the control they have (ease or difficulty) of performing a specific behaviour [11,14]. According to this model, human attitudes formed by behavioural attitudes, subjective norms and perceived behavioural control, determine intention and influence human behaviour (interactions, husbandry and management) which has been showed, in other livestock industries, to have implications for animal behaviour, productivity and welfare [12,15–21].

Understanding the underlying beliefs that underpin farmers’ management behaviour, as well as main barriers for adoption, have the possibility to create broad industry impact. With this information, it is possible to develop adequate educational programs to promote attitudinal change, and thus increase the implementation of improved welfare practices with the goal of achieving sustained practice change in the sheep industry. This present study is part of a larger research investigating farmer attitudes and sheep welfare outcomes. This paper reports on the relationship between farmer attitudes, management behaviour and sheep welfare and builds on a published study by Munoz et at., [22] that reports details on the on-farm welfare of extensively managed sheep.

## Materials and methods

### Farms and visits

To determine relationships between farmer attitudes, management behaviour and ewe welfare, questionnaire interviews with sheep farmers and two visits to their property to perform animal welfare assessments were conducted. This study was approved by the University of Melbourne Animal and Human Ethics Committees, ethical review numbers 1613838 and 1646392 respectively. In total, 32 commercial sheep farms, located in the high rainfall (> 600 mm) and wheat-sheep regions (300–600 mm) of Victoria, Australia were involved in this study. Farmers were recruited through advertisements in industry magazines, by engaging with local consultants and their groups, by advertising in industry conferences or through nomination by other farmers. Eligible farms had to have a self-replacing ewe flock, spring lambing and contain a minimum of 400 breeding ewes. This lower limit was based on preliminary results from focus group discussions with Victorian farmers [23].

### Questionnaires

Two questionnaires were used; a farmer attitude questionnaire and a management questionnaire, both completed by the main person in charge of the animals, either the owner or the farm manager. The attitude questionnaire measured general attitudes towards sheep, sheep management and husbandry practices, using three types of attitude statements, based on attitudes towards the behaviour, subjective norms and perceived behavioural control. The questionnaire contained 88 statements in total and was developed based on results from two focus group discussions [23]. Using a five-point Likert scale, farmers were asked to indicate their level of agreement to the statements, the level of importance or perceived difficulty of performing and activity e.g. score 1 = not at all agree/not at all important/not at all difficult to score 5 = strongly agree/very important/very difficult. Questions were both positive and negative, but responses were re-coded so that a high score indicated that the farmer held a positive attitude towards sheep and sheep management. Several statements on a specific topic were used to measure consistent beliefs, which allows the identification of a person’s attitude towards a specific topic [12]. Thus, the attitudes that farmers held regarding sheep and management practices were used to establish their attitudes towards these aspects.

The management questionnaire involved a total of 51 questions on demographics, farm characteristics, animal numbers, labour units, production type, self-reported sheep husbandry and management practices, and perceived ‘barriers’ to best practice. Specifically, famers were asked if they performed the following management practices: daily monitoring of the ewe flock, monitoring of feed on offer, body condition scoring of ewes, ultrasound pregnancy diagnosis annually, keep mortality records, keep productive records, perform internal parasite egg count before deworming sheep, provide sheep with some sort of shelter (e.g. trees) and whether they manage ewes according to their nutritional needs. This section was conducted in an interview-style, where the lead researcher (CAM) and the farmer discussed the questions. This allowed the farmer to clarify anything he/she was uncertain of, and for the researcher to follow up with any questions for clarification. The completion of the questionnaires took from 90 to 180 min, depending on the level of discussion that occurred, both questionnaires were conducted a day before the first welfare assessment. Farmers were also asked to complete a stock tally form to provide details on their sheep numbers from 1^st^ of July 2016 to 30^th^ of June 2017.

### Animals and welfare assessments

Welfare assessments were conducted at two-time points. Visits were arranged to coincide with mid-pregnancy and weaning 2016/17. Sheep were managed under extensive commercial conditions, in year-round outdoor systems.

The assessments were performed using a holding pen and a single-file race within the regularly-used sheep yards of each farm. At each visit, farmers were asked to provide a random sample of 100 ewes, aged from two to five years. The ewes were examined using five animal-based welfare measures: body condition, fleece condition, number of skin lesions, faecal soiling and lameness. The assessment criteria for the welfare measures are presented in Table 1. In addition, the number of animals in need of further care (e.g. such as injured or sick sheep) were recorded at each farm at both time periods. Further care was defined as any sick or injured ewe that would benefit from further inspection and/or intervention. This included, but was not limited, to poor body condition, poor fleece condition, severe injuries (e.g. fresh, bleeding and ≥10 cm) and severe lameness (e.g. score 2 or more). Further details on the on-farm welfare of the ewes is reported in [22].

**Table 1:** Animal-based welfare measures used to assess ewe welfare.

Welfare measures	Assessment criteria
Body condition score	Scored on a 5-point scale, using a quarter-unit precision [24,25] (1) Emaciated. Dorsal spinous and transverse processes are sharp and prominent. (2) Thin. Dorsal spinous processes are still prominent, but not as sharp. Transverse processes rounder on edges. (3) Average. Spinous and transverse processes are smoother and less prominent. (4) Fat. Considerable pressure is needed to feel dorsal spinous processes. Transverse processes cannot be felt (5) Obese. Dorsal spinous and transverse processes cannot be felt.
Fleece condition	Scored on a 3-point scale: (0) Good fleece condition, when parted, the fleece has no lumpiness or signs of ectoparasites (1) Some fleece loss, small shedding or bald patches ≤ 10 cm diameter. When parted, the fleece may have some lumpiness or scurf, little evidence of ectoparasites (2) Significant fleece loss with bald patches of greater than 10 cm in diameter, clear evidence of ectoparasite [26]
Skin lesions	Assessed by recording number, location and severity of the skin lesions. Lesions were classified as cuts, open wounds, old wounds or scars and abscesses.
Dag score	Scored on a 6-point scale: (0) No evidence of faecal soiling (1) Very light soiling on the breech area (2) Moderate dag on the breech area extending ventrally (3) Severe dag predominantly on the breech area, extending ventrally and dorsally over the tail some soiling and dag around anus (4) Excessive dag on the breech area and on the hind legs (5) Very severe dag on the breech area and on the hind legs or below the level of the hocks [27]
Lameness	Scored on a 4-point scale: (0) Not lame (1) Clear shortening of stride with obvious head nodding or flicking as the affected limb touches the floor (2) Clear shortening of stride with obvious head nodding and not weight-bearing on affected limb whilst moving (3) Reluctant to stand or move [26]

Resource-based measures were collected by assessing the farms’ yards. The quality of the yards was subjectively scored from 0 to 7 by the lead researcher. Measures taken into consideration were; yard design (the ease of moving sheep from pens to the race), condition of the construction materials, quality of gates (ease of closing and opening gates), quality of floor (even floor), presence of roof, adequate wide and adequate length of the race to work with sheep.

### Statistical analysis

The survey was analysed using qualitative and quantitative analysis. The questionnaires consisted of continuous data (e.g. farm hectares, sheep numbers, labour units, etc) and categorical, ordinal (attitude statements using a five-point Likert scale) and binomial data (management questions where farmers responded yes/no). Fisher’s exact tests (FET) were used to compare differences between management practices and ewe breeds (wool breeds vs meat breeds) and locations (high rainfall vs wheat-sheep regions).

Farmers’ self-reported management behaviour and responses to the attitude statements were analysed by categorical principal component analysis, which is the non-linear equivalent of PCA. This procedure is available in SPSS as CATPCA. As with PCA, this technique reduces a set number of variables to a small number of new variables, known as principal components (or dimensions). Overall, CATPCA and PCA are very similar in objective, method, results, and interpretation. However, one of the main advantages of CATPCA is that can be used for nominal and ordinal variables. Likert-type scale values are not truly numeric, because intervals between consecutive categories cannot be assumed to be equal. Thus, to obtain the component scores, CATPCA analysis iteratively computes the component scores from the data itself, using an optimal scaling process to quantify the variables according to their analysis level [28].

Spearman’s Rank correlation analyses were subsequently used on the CATPCA components scores to examine relationships between management and welfare outcomes and farmers general attitudes to sheep and welfare outcomes. Correlation analyses were also used to examine relationships between farm characteristics and welfare outcomes and to examine relationships between the attitude subscales (attitude towards the behaviour, subjective norms and perceived behavioural control). Correlation values were classified as strong if coefficients were ≥ 0.6 and moderate if between 0.3 and 0.59 [29]. Corrections to P values such as the Bonferroni correction were not conducted in this study to minimise the risk of type II errors [30–33].

Logistic regression analyses (stepwise selection) were used to determine which of the independent variables (attitudes towards the behaviour, subjective norms and/or perceived behavioural control) had a statistically significant effect on farmer management behaviour. In addition, ordinal logistic regression analyses were performed to determine which of the background factors; age, work experience and level of education (independent variables) were significant predictors of the attitude subscales; attitudes towards the behaviour, subjective norms and/or perceived behavioural control (dependent variables). Data were analysed using the statistical programs SPSS 16.0 and SAS 9.4 statistical package.

## Results

### Relationship between management behaviour and sheep welfare

From the 32 farms in the study, fourteen farms were meat-focused enterprises (44%), twelve were meat-wool enterprises (38%), and six were wool focused enterprises (18%). Overall, common practices among sheep farmers were visual monitoring of both the ewe flock and feed on offer. All farmers reported that monitoring of the flock was done visually, from a distance while driving around the farm, and/or when feeding or moving sheep. However, the frequency of monitoring varied depending on the reproductive stage of the ewes. During lambing periods, 87.5% of farmers monitored their flocks twice daily (n = 20) or daily (n = 8). During non-lambing periods, farmers monitored their flock once weekly (66%, n = 21), every second day (16%, n = 5), fortnightly (16%, n = 5) or monthly (3%, n = 1). There was no statistical difference between meat-breed farmers and wool-breed farmers. Visual monitoring of feed on offer was a common activity and did not differ between meat-breed farmers and wool-breed farmers or across regions. While less common, a total of 19% (n = 6) of the farmers reported the use of more technical approaches such as industry guidelines, rulers/sticks or the advice of agronomists to measure pasture availability and quality. When asked about why some farmers did not monitor their flock or pasture every day (during the lambing period), the most common response was to not cause mismothering (31%). When asked about why some farmers did not monitor their flock or pasture every day(outside the lambing period), the most common responses were that they were busy with other activities or had other priorities (34%).

A total of 23 (72%) farmers self-reported that they checked the body condition of their ewes, and this was most commonly among meat-breed farmers than wool farmers (*P* = 0.02, FET). However, when asked to describe how they checked the body condition of their ewes, 14 (61%) farmers out of the 23 reported that they did this by physically touching the back of the ewes, while nine (39%) out of the 23 only did so visually from the distance. The latter group of farmers were re-coded as ‘not performing this activity’ for further analysis. Lack of time or not enough labour were cited as the most common reasons farmers did not physically condition score their ewes. Other descriptive reasons included farmers not wanting to do it or not appreciating the value of it.

Deworming sheep was usually performed once a year (41%, n = 13) or twice a year (34%, n = 12) and most commonly before lambing (44%) or during summer (41%), but most farmers (72%) agreed this activity was highly influenced by environmental conditions. A total of 66% (n = 21) of farmers performed egg count before deworming sheep. No differences were observed between flock type or region. Ultrasounds pregnancy diagnosis was more commonly performed on meat-breed flocks than wool flocks (*P* = 0.02, FET). Overall, a total of 22 (69%) of farmers scanned for pregnancy every year, six farmers (19%) scanned only in particular years and four farmers (13%) had never scanned. The main reason farmers decided not to perform ultrasound pregnancy diagnosis annually was cost (25%, n = 8). Other reasons listed were; poor facilities, another activity to do, farmers not appreciating the benefits or not willing to change their practices, not being considered a priority and ‘indolence’.

Record keeping was a less common activity and not influenced by flock type or region. Farmers were more likely to keep records of marking (50%) and conception rates (47%), than weaning (44%) and mortality records (41%). While 41% of farmers (n = 13) self-reported to keep mortality records, only 34.4% (n = 11) of farmers provided accurate, or close to accurate, stock tally records. Although mortality records were not usually accurate, a total of 47% (n = 15) of farmers reported that the main cause of ewe mortality was dystocia. Other causes were related to low body condition, internal parasites and age. Farmers estimated that their annual ewe mortality was 2.7% (range from 1 to 5%). However, according to the stock tally records, annual ewe mortality rate was 4.7% (ranged from 0.2 to 14.4%). Comparing farmer responses in the questionnaire and the stock tally data, six farmers overestimated their mortalities while 16 (50%) underestimated their mortality rates. Lack of time was the most common reason for not keeping records. Other reasons focused on the difficulty of measuring, not wanting to know the answer, not interested or not seeing the value in knowing mortalities rates, some quotes included ‘it’s difficult to keep accurate records’, ‘it’s difficult to work in the office when you are tired from working sheep’, ‘you don’t want to keep remembering how much you have lost’, ‘The information doesn’t change my operation’, and ‘(you) can’t make a dead sheep alive’.

Overall, industry engagement in the study group was high. A total of 69% (n = 22) of farmers were active members of industry groups. The most common industry group membership was to BestWool/BestLamb (Australian sheep farmers industry network) with 50% of the farmers registered. Attendance to workshops or field days was even more common among these farmers as 81% (n = 26) of participants reported that they attended at least one workshop within 12 months prior to this study.

Five specific questions on management were used for analyses between self-reported management behaviour and ewe variables. The questions were reduced to one component using CATPCA. The component accounted for 48% of the variance (Cronbach’s alpha 0.73) and included the following questions ‘Do you perform egg count before deworming?’, ‘Do you perform ultrasound pregnancy diagnosis annually?’, Do you manage ewes according to nutritional needs? ‘Do you condition score ewes’? Do you measure feed on offer? This component reflects active sheep management and was labelled accordingly. The CATPCA component score was subsequently used to examine relationships between management and welfare outcomes at mid-pregnancy and weaning with results presented in Table 2. Results disclosed moderate negative relationships between *Active Management* and lameness and ewes that needed further care at mid-pregnancy. Also, moderate negative relationships were observed between *Active Management* and skin lesions and ewes that needed further care at weaning. Overall these results indicate that farmers that performed more management activities had fewer lame ewes at mid-pregnancy, fewer ewes with skin lesions at weaning and fewer ewes that needed further care after both assessments.

**Table 2:** Spearman’s rank correlations between management and welfare outcomes.

Welfare measures	Active Management	
	Mid-pregnancy	Weaning
BCS		
≤ 2.25 (low)	-0.05	-0.04
2.5–3.5 (adequate)	0.07	0.05
≥ 3.75 (fat)	-0.01	0.03
Fleece condition		
score 1–2	-0.03	-0.02
Skin lesions		
(count)	0.16	**-0.44**
Dag score		
score 4–5	0.01	0.12
Lameness		
score 1–3	-0.38*	-0.23
Further Care		
(count)	-0.47**	-0.50**

*Correlation significant at *p* ≤ 0.05,

** Correlation significant at *p* ≤ 0.01.

### Relationship between farm characteristics and sheep welfare

There was large variation in the study group in relation to farm characteristics. The average ewe flock size was 2,771 (± 2770, n = 32), however there was a wide range in ewe numbers (431–9,400) and farm sizes (200–3200 hectares). Details on farm flock sizes and sheep breeds are presented in Table 3.

**Table 3:** Farm demographics according to enterprise, flock size and breed. The range of the ewe flock sizes is presented in parentheses.

Enterprise	Farms	Average flock size	Breed
Meat	14	2,770 (500–9000)	^#^Composite, Poll Dorset Highlander, Corriedale
Meat-wool	12	2,246 (431–4411)	Merino, ^^^Merino first-cross, Composite, Dohne
Wool	6	2,091 (1075–9400)	Merino

^#^Composite breeds were mainly Coopworths (Border- Romney, F3 generation progeny).

^Merino first-cross ewes refer to the offsprings of Merino ewes with Border Leicester rams

Labour units averaged 1.7 Full-time employees (FTE) and ranged between 0.5 to 3.5 FTE. The quality of the yards also varied significantly with an average score of 4.5 (± 1.8, n = 32) and ranged from 1 to 7. Results disclosed that *yards*, *hectares*, *flock size and sheep*:*labour ratio* were correlated with welfare outcomes. As presented in Table 4, moderate negative relationships were found between *yards*, *hectares and flock size* and fat ewes and ewes that needed further care at weaning. *Flock size* also showed moderate negative relationships with lameness at weaning. At mid-pregnancy, *sheep*:*labour ratio* had a moderate negative relationship with inadequate fleece condition. While not statistically significant, *sheep*:*labour ratio* tended to be negatively associated with ewes that needed further care at weaning (r = -0.33, *P* = 0.06). Moderate positive relationships were also disclosed between *yards* and *flock size* (r = 0.58, *P* = 0.000), between *yards* and *hectares* (r = 0.50, *P* = 0.001) and between *yards* and *sheep*:*labour ratio* (r = 0.46, *P* = 0.001).

**Table 4:** Spearman’s rank correlation between farm characteristics and welfare outcomes.

Welfare measures	Yards	Hectares	Flock size	Sheep:labour
MID- PREGNANCY				
BCS				
≤ 2.25 (low)	-0.23	-0.11	-0.09	-0.10
2.5–3.5 (adequate)	0.22	0.23	-0.02	-0.08
≥ 3.75 (fat)	-0.04	-0.27	0.02	0.15
Fleece condition				
score 1–2	-0.12	-0.23	-0.32	-0.36*
Skin lesions				
(count)	0.13	0.31	0.09	-0.19
Dag score				
score 4–5	-0.05	0.18	0.19	0.07
Lameness				
score 1–3	-0.04	-0.20	0.01	0.25
Further Care				
(count)	-0.05	-0.27	-0.05	-0.02
WEANING				
BCS				
≤ 2.25 (low)	0.21	0.31	0.22	0.09
2.5–3.5 (adequate)	-0.08	-0.14	-0.03	0.09
≥ 3.75 (fat)	-0.34*	-0.42*	-0.47**	-0.44*
Fleece condition				
score 1–2	0.09	0.03	0.29	0.32
Skin lesions				
(count)	-0.07	-0.13	0.13	0.22
Dag score				
score 4–5	-0.08	0.08	-0.01	-0.09
Lameness				
score 1–3	-0.28	-0.29	0.37*	-0.29
Further Care				
(count)	-0.51**	-0.40*	-0.52**	-0.33

*Correlation significant at *p* ≤ 0.05,

**Correlation significant at *p* ≤ 0.01.

### Relationship between general attitudes towards sheep and sheep welfare

All farmers considered themselves to be responsible for the welfare of their animals and 84% recognised that how people handle ewes will affect their fearfulness. In the attitude questionnaire, there were six statements focused on general attitudes about sheep. These were reduced to two components using CATPCA and varimax rotation. Component one included the statements ‘ewes are NOT stubborn’ ‘ewes are NOT frustrating to work with’ and ‘I think ewes are NOT annoying’. This component accounted for 40.0% of the variance (Cronbach’s alpha 0.70) and was labelled ‘Positive attitudes towards sheep’. The second component included the statements, ‘Farm animals have feelings like people have feelings’, ‘Ewes have a gentle nature’ and ‘Ewes are intelligent’. This second component accounted for 33.6% of the variance (Cronbach’s alpha 0.61) and was labelled ‘Positive attitudes towards sheep as sentient animals.’ Spearman’s Rank correlation analyses between the CATPCA components scores and welfare outcomes disclosed moderate, negative relationships between *Positive attitudes towards sheep* and lameness and ewes that needed further care at weaning (Table 5). Moderate positive correlations were observed between *Positive attitudes towards sheep* and fat ewes at mid-pregnancy. In addition, moderate negative relationships were found between *Positive attitudes towards sheep as sentient animals* and ewes with low body condition and skin lesions. In general, these results indicate that farmers with more positive attitudes towards sheep had fewer lame ewes and fewer ewes in need of further care at weaning. Also, farmers with more positive attitudes towards sheep had fewer thin ewes, more fat ewes and fewer ewes with skin lesions at mid-pregnancy. No significant correlations were found between positive attitudes towards sheep and active management, although a tendency of a moderate positive relationship was disclosed at weaning (r = 0.33 p = 0.07).

**Table 5:** Spearman’s rank correations between general attitudes towards sheep and welfare outcomes.

Positive Attitudes Towards		
Welfare measures	Sheep	Sheep as sentient animals
MID-PREGNANCY		
BCS		
≤ 2.25 (low)	-0.23	-0.38*
2.5–3.5 (adequate)	0.14	0.18
≥ 3.75 (fat)	0.41*	0.33
Fleece condition		
score 1–2	0.01	-0.05
Skin lesions		
(count)	-0.34	-0.40*
Dag score		
score 4–5	-0.15	-0.09
Lameness		
score 1–3	0.21	0.23
Further Care		
(count)	-0.04	0.02
WEANING		
BCS		
≤ 2.25 (low)	-0.01	0.19
2.5–3.5 (adequate)	0.07	-0.18
≥ 3.75 (fat)	-0.24	-0.09
Fleece condition		
score 1–2	-0.07	0.02
Skin lesions		
(count)	-0.15	0.07
Dag score		
score 4–5	-0.09	0.07
Lameness		
score 1–3	-0.42*	-0.18
Further Care		
(count)	-0.36*	-0.24

*Correlation significant at *p* ≤ 0.05

There were nine statements on farmers general attitudes relating to handling and moving sheep. These were reduced to two components using CATPCA and varimax rotation. The first component accounted for 31.8% of the variance (Cronbach’s alpha 0.74) and included the following statements: ‘The way people handle sheep impact ewes’ fearfulness’, ‘Moving sheep is an easy task’, ‘Sheep are easy to train to a routine’, ‘If someone is roughly handling my sheep, I would intervene’, ‘The best way to move sheep is by not rushing them’ and ‘I am good at handling sheep’. This component was labelled as ‘Positive attitudes towards handling and moving sheep’. The second component accounted for 28.2% of the variance (Cronbach’s alpha 0.68) and included the statements: ‘Using dogs is the best way to move sheep’, ‘Using dogs is not stressful for sheep’ and ‘Sheep are easy to handle’. This second component was labelled as ‘Positive attitudes towards using dogs’. Spearman’s rank correlation analyses between the CATPCA component scores and welfare outcomes showed moderate negative relationships between *Positive attitudes towards handling and moving sheep* and lameness at weaning (Table 6). Moderate positive relationships were also observed between *Positive attitudes towards using sheep dogs* and lameness at mid-pregnancy. While not statistically significant, *Positive attitudes towards using sheep dogs* tented to be negatively associated with adequate BCS at mid-pregnancy (r = -0.31, *P* = 0.09). These results indicate that farmers with more positive attitudes towards handling and moving sheep, and with less positive attitudes towards using sheep dogs, had fewer lame ewes and tended to have more ewes in adequate body condition.

**Table 6:** Spearman’s rank correations between general attitudes towards handling sheep and welfare outcomes.

Positive Attitudes Towards		
Welfare measures	Handling and moving sheep	Using sheep dogs
MID-PREGNANCY		
BCS		
≤ 2.25 (low)	-0.14	0.08
2.5–3.5 (adequate)	0.16	-0.31
≥ 3.75 (fat)	0.04	0.23
Fleece condition		
score 1–2	0.25	-0.33
Skin lesions		
(count)	-0.10	-0.22
Dag score		
score 4–5	0.07	-0.05
Lameness		
score 1–3	-0.04	0.39*
Further Care		
(count)	-0.24	0.30
WEANING		
BCS		
≤ 2.25 (low)	0.24	-0.04
2.5–3.5 (adequate)	-0.27	-0.09
≥ 3.75 (fat)	-0.09	0.01
score 1–2	-0.04	-0.01
Skin lesions		
(count)	0.01	0.17
Dag score		
score 4–5	-0.08	-0.07
Lameness		
score 1–3	-0.57**	0.10
Further Care		
(count)	-0.26	0.06

*Correlation significant at *p* ≤ 0.05,

**Correlation significant at *p* ≤ 0.01.

### Relationships between farmer attitudes and farmer self-reported management behaviour

Only a few attitude subscales were significantly associated with farmer management behaviour (6 out of 18 variables analysed). However, the relationships observed tended to be in the expected direction. A positive self-evaluation of the behaviour (attitude towards behaviour, labelled as ‘attitude’), a perceived positive evaluation of the behaviour by others (subjective norms, labelled as ‘SN’) or the belief that the behaviour can be realised (perceived behavioural control, labelled as ‘PBC’) were predictive of management behaviour by sheep farmers. Only results significant at *p* ≤ 0.05 are reported in Table 7. Condition scoring ewes, ultrasound pregnancy diagnosis and testing for egg count before deworming sheep were all positively predicted by the attitude towards the behaviour, which refers to the importance/value farmers place on these management practices. Keeping mortality records and provision of shelter were both positively predicted by subjective norms. Managing ewes according to nutritional needs was negatively predicted by perceived behavioural control (Odds ratio = 0.26, *P* = 0.01), which indicates that when farmers perceived this activity to be difficult, they were less likely to perform this activity.

**Table 7:** Logistic regression analysis for farmer attitudes about management and farmer self-reported management behaviour (n = 32).

Management behaviour	Attitude Variable	β coefficient	Standard error	P-value	Odds ratio	95% C.I for odds ratio	
						Lower	Upper
Condition scoring ewes	Attitude	0.86	0.39	**0.03**	2.37	1.12	5.04
Ultrasound pregnancy diagnosis	Attitude	1.16	0.5	**0.02**	3.19	1.21	8.41
Mortality records	SN	1.13	0.44	**0.01**	3.04	1.28	7.24
Egg count before deworming	Attitude	1.06	0.4	**0.01**	2.88	1.31	6.29
Provision of shelter at winter	SN	2.13	0.98	**0.03**	8.41	1.24	57.29
Manage according to nutritional needs	PBC	-1.35	0.55	**0.01**	0.26	0.09	0.76

The variable ‘Attitude’ refers to attitudes towards the behaviour, ‘SN’ refers to subjective norms and ‘PBC’ refers to perceived behavioural control. Example of questions concerning attitudes towards the behaviour: How important is it to (e.g. condition score ewes)? Example of questions concerning subjective norms: How important does your trusted advisor believe it is to (e.g. body condition score ewes)? Example of questions regarding perceived behavioural control: How difficult is it for you to (e.g. body condition score ewes)?

Overall, there was a low perceived difficulty for all management practices, with farmers usually rating the activities ‘not at all difficult’ or ‘slightly difficult’. However, while management practices were usually perceived as not difficult, most farmers agreed with the statement ‘No matter how hard I try, a constant number of ewes will always die’ (72%) and ‘Weather will influence lamb survival more than any management decisions I make’ (53%).

Significant inter-correlations between some attitude subscales were found across the behaviours. Strong to moderate positive correlations were found between positive attitude towards managing ewes according to nutritional needs and positive attitudes towards ultrasound diagnosis (r = 0.62, *P* ≤ 0.01), and between positive attitudes towards monitoring feed on offer and keeping mortality records (r = 0.55, *P ≤ 0*.*01*). Similarly, positive correlations were obtained between perceived difficulty of performing ultrasound diagnosis annually and perceived difficulty of managing ewes according to nutritional needs (r = 0.63, *P* ≤ 0.01), and between perceived difficulty of monitoring feed on offer and monitoring the flock (r = 0.47, *P* ≤ 0.01). Significant negative correlations were also observed between perceived difficulty of managing ewes according to nutritional needs and (1) positive attitudes towards managing ewes according to nutritional needs (r = -0.48, *P* ≤ 0.01), (2) positive attitudes towards performing egg count before deworming sheep (r = -0.42, *P* = 0.02), (3) positive attitudes towards monitoring feed on offer (r = -0.41, *P* = 0.02) and (4) positive attitudes towards ultrasound pregnancy diagnosis (r = -0.38, *P* = 0.03).

### Relationships between background factors and farmer attitudes

Most farmers/managers in this study were male (n = 30) and their average age was 52 (16.5 ± SD) years. The educational level in the study group ranged from ‘secondary school’ (22%, n = 7) to ‘university degree’ (56%, n = 18) and the average number of years working with sheep was 26.7 (17.7 ± SD) years.

The ordinal logistic regression analyses showed that farmers’ background factors had some role in predicting farmers attitudes concerning sheep management (Table 8). *Work experience* (WE) predicted perceived behavioural control concerning condition scoring ewes, keeping mortality records and provision of shelter at winter. All these relationships were positive, which mean that farmers with more years of work experience perceived condition scoring of ewes, keeping mortality records and provision of shelter at winter to be more difficult activities. *Work experience* also predicted behavioural attitudes towards managing ewes according to nutritional needs. This relationship was negative meaning that farmers with more years of work experience tended to perceive this activity to be less important. *Level of education* predicted perceived behavioural control concerning condition scoring ewes and mortality records. The direction of these relationships was negative meaning that farmers with higher the level of education perceived condition scoring of ewes and keeping mortality records to be less difficult activities. While not statistically significant, *level of education* also tented to predict subjective norms concerning ultrasound pregnancy diagnosis (the direction of the relationship was positive). *Age* predicted perceived behavioural control concerning body condition scoring of ewes, keeping mortality records and tended to predict provision of shelter at winter (Odds ratio = 0.93, *P* = 0.07). The direction of these relationships was negative which means that older farmers perceived the aforementioned activities to be less difficult. No background factors were predictive of farmers attitudes concerning performing egg count before deworming sheep. Results significant at *p* ≤ 0.05, or close to significant, are reported in Table 8.

**Table 8:** Ordinal logistic regression analyses for farmer attitudes about management behaviour and farmers background factors (n = 32).

Management behaviour	Attitude Variable	Background factor	β coefficient	P-value	Odds ratio	95% C.I for odds ratio	
						Lower	Upper
Condition scoring ewes	PBC	Age	-0.16	**0.00**	0.85	0.76	0.95
		WE	0.13	**0.01**	1.14	1.03	1.26
		Education	-3.57	**0.01**	0.03	0.01	0.42
Ultrasound pregnancy diagnosis	SN	Education	1.99	*0*.*08*	7.38	0.78	70.14
Mortality records	PBC	Age	-0.10	**0.05**	0.90	0.81	1.00
		WE	0.13	**0.01**	1.14	1.03	1.26
		Education	-2.44	**0.03**	0.87	0.01	0.79
Provision of shelter at winter	PBC	WE	0.09	**0.02**	1.09	1.01	1.19
		Age	-0.08	*0*.*07*	0.93	0.85	1.00
Manage according to nutritional needs	Attitude	WE	-0.10	**0.04**	0.90	0.82	0.99

The variable ‘Attitude’ refers to attitudes towards the behaviour, ‘SN’ refers to subjective norms and ‘PBC’ refers to perceived behavioural control. Age refers to the farmers or farm managers age. Work experience (WE) refers to the years of experience working with sheep of the farmers or farm managers. Education refers to the level of education of the farmers or farm managers.

## Discussion

This study hypothesised that farmer attitudes relate to management behaviour and management behaviour relates to the on-farm welfare of extensively managed sheep. To our knowledge, this is the first study investigating these relationships in extensive sheep farming systems. The main results of this study indicate relationships between farmer attitudes and management behaviour, as well as relationships between management behaviour and ewe welfare outcomes, consistent with the Theory of Planned Behaviour and the HAR model [12,13] and in agreement with previous studies in the pig, poultry and dairy industries [21,34–37].

When combining the qualitative and quantitative analyses, behavioural attitudes (attitudes towards specific management behaviours) and perceived behavioural control (perceived barriers to performing the behaviour) emerged as the two main drivers underpinning farmer management behaviour. While the results from the regression analyses indicated that effect of perceived behavioural control in farmer management behaviour was limited, the fact that most farmers ranked management practices as being ‘not at all difficult’ or ‘slightly difficult’ may have influenced these results. When participants were further asked about why they or other farmers did not perform improved practices, comments were mainly focused on not having enough time to perform the activity or practices not being able to influence the outcome. This suggests that the perceived difficulty of performing an activity was, indeed, an important barrier to farmers. This also indicates that sometimes there was a disconnect between specific management practices and the impact or value they can have on sheep welfare. Overall, these results demonstrate the potential to alter farmer behaviour by modifying the key attitudes and barriers that underpin these behaviours.

By modifying one key behaviour it may be possible to modify other farm management practices. Significant inter-correlations between some of the attitude subscales (attitudes towards the behaviour and perceived behavioural control) were observed in this study, which may represent a general farmer attitude towards the performance of management behaviours [34]. Consistency of attitudes towards management behaviours suggests that if farmers have a positive attitude towards one key management behaviour (e.g. ultrasound pregnancy diagnosis), they are likely to have positive attitudes towards similar behaviours (e.g. manage ewes according to nutritional needs). These findings are consistent with the HAR model [12] and add to the existing literature for other livestock industries in which some consistent patterns of correlations between the attitude subscales and several stockperson behaviours have been identified [15,18,20,38,39]. Practically, this suggests that by modifying one key behaviour it may be possible to modify other practices. This could be used to encourage change in management, however, there is a risk of a reverse relationship: if a farmer does not believe in the value of one management practice, they may stop believing in the value of performing others as well.

Active management was associated with positive sheep welfare outcomes, as indicated by fewer ewes with skin lesions, fewer lame ewes and fewer ewes in need of further care. Overall, visual monitoring of the flock and feed on offer were common management practices among farmers, while body condition scoring, performing internal parasite egg count before deworming sheep, ultrasound pregnancy diagnosis and records keeping were less common activities. It is important to consider that while most farmers reported that they checked the body condition of their sheep, 39% of farmers misunderstood how to effectively perform this activity reporting that body condition scoring was performed visually from the distance. For records keeping, engagement was limited and the accuracy with which farmers could estimate issues on the farm was low, with stock tally data usually not corresponding with the estimated ewe mortality rates provided by the farmer, and mortality rates were frequently underestimated. If data are not recorded and not reliable, then it is difficult to estimate the productivity, health and welfare issues of the farm to make interventions accordingly. Thus, improved methods to monitor the flock and keep accurate records are needed. Providing farmers with easily accessible and practical alternatives to improve data collection, such as the use of electronic identification (EID) systems, would be a valuable and relatively low-cost alternative to benefit welfare and productivity. However, the implementation of any strategy should be combined with programs targeting farmer attitudes in order to achieve a sustained change in farmer behaviour.

Welfare compromise did not necessarily increase with farm size. Larger farms in this study had yards in better condition and had fewer fat ewes, fewer lame ewes and fewer ewes in need of further care at weaning. While not tested in this study, previous studies have demonstrated that larger farms (>5,000 head) have the ability to access more resources (e.g. veterinary assistance) [40] and stockpeople are more likely to have formal training [41]. These relationships were more evident at weaning as these welfare outcomes were probably exacerbated by seasonal and physiological variation at this period [4,42].

General attitudes towards sheep were associated with welfare outcomes. Positive attitudes towards handling and moving sheep were associated with fewer lame ewes. However, it is important to consider that direct evidence on the effects of handling and moving sheep were not measured in this study. In addition, the attitudes and the behaviours of farm contractors, who often handle sheep during routine husbandry procedures, were not examined. For further studies, it would be valuable to assess both farmer and contractors’ attitudes and behaviour during routine husbandry procedures such as lamb marking, weaning or ultrasound diagnosis, to better understand the human-animal relationship in extensive systems.

**Fig 2:**
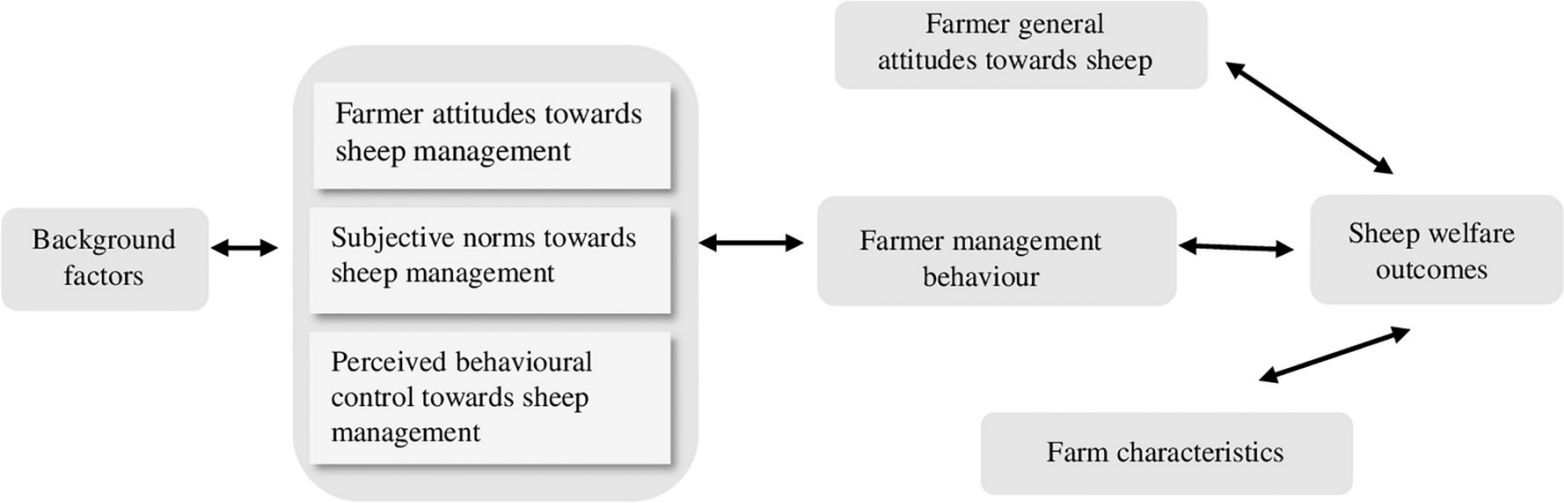
Proposed farmer-sheep relationships in extensive farming conditions.

The relationship observed between level of education and farmers attitudes towards management behaviour demonstrates a key role for education in farmer behaviour. This is further supported by previous research that has demonstrated that education programs designed to promote attitudinal change have been successful in the pork [12,39] and dairy [43] industries. Overall, the main findings of this study indicate relationships between farmer attitudes and management behaviour, relationships between management behaviour and ewe welfare outcomes and relationships between background factors and farmer attitudes. Based on our main findings, and the HAR Model [12], Fig 2 illustrates the proposed farmer-sheep relationships in extensive farming conditions.

Although this present study does not permit causal inferences to be made, if education programs are able to improve farmer attitudes and behaviour as well as sheep welfare, then causality could be investigated. Based on our findings, a potential intervention in the sheep industry may involve the implementation of an education program intended to improve farmer self-evaluation of the behaviour (behavioural attitudes) and their perceived control regarding the behaviour. The focus should be put on flock monitoring, BCS and records keeping as these management practices influence welfare and productivity despite location and type of enterprise.

## Conclusions

The main results of this study indicate relationships between farmer attitudes and management behaviour, as well as relationships between management behaviour and ewe welfare outcomes. Overall, farmers that held more positive attitudes towards sheep management were more likely to have an active management style and had fewer ewes in need of further care at mid-pregnancy and weaning. Both behavioural attitudes and perceived behavioural control were predictive of farmer behaviour, and these key attitudes should be addressed in future education strategies aimed at promoting practice change. Further research needs to provide evidence of causal relationships and determine the effectiveness of education programs in achieving attitudinal and sustained practice change in the sheep industry.

## Supporting information

S1 Appendix CATPCA components data supporting information. (XLSX)

S2 Appendix Regression analyses data supporting information. (XLSX)
